# Revolutionizing lymph node metastasis imaging: the role of drug delivery systems and future perspectives

**DOI:** 10.1186/s12951-024-02408-5

**Published:** 2024-03-29

**Authors:** Ze-Min Cai, Zi-Zhan Li, Nian-Nian Zhong, Lei-Ming Cao, Yao Xiao, Jia-Qi Li, Fang-Yi Huo, Bing Liu, Chun Xu, Yi Zhao, Lang Rao, Lin-Lin Bu

**Affiliations:** 1 State Key Laboratory of Oral & Maxillofacial Reconstruction and Regeneration, Key Laboratory of Oral Biomedicine Ministry of Education, Hubei Key Laboratory of Stomatology, School & Hospital of Stomatology, Wuhan University, Wuhan, 430072 China; 2 Department of Oral & Maxillofacial Head Neck Oncology, School & Hospital of Stomatology, Wuhan University, Wuhan, 430079 Hubei China; 3 Department of Prosthodontics, School and Hospital of Stomatology, Wuhan University, Wuhan, China; 4 School of Dentistry, The University of Queensland, Brisbane, QLD 4066 Australia; 5 Institute of Biomedical Health Technology and Engineering, Shenzhen Bay Laboratory, Shenzhen, 518132 China

**Keywords:** Drug delivery systems, Lymph node metastasis, Imaging, Computed tomography, Magnetic resonance

## Abstract

The deployment of imaging examinations has evolved into a robust approach for the diagnosis of lymph node metastasis (LNM). The advancement of technology, coupled with the introduction of innovative imaging drugs, has led to the incorporation of an increasingly diverse array of imaging techniques into clinical practice. Nonetheless, conventional methods of administering imaging agents persist in presenting certain drawbacks and side effects. The employment of controlled drug delivery systems (DDSs) as a conduit for transporting imaging agents offers a promising solution to ameliorate these limitations intrinsic to metastatic lymph node (LN) imaging, thereby augmenting diagnostic precision. Within the scope of this review, we elucidate the historical context of LN imaging and encapsulate the frequently employed DDSs in conjunction with a variety of imaging techniques, specifically for metastatic LN imaging. Moreover, we engage in a discourse on the conceptualization and practical application of fusing diagnosis and treatment by employing DDSs. Finally, we venture into prospective applications of DDSs in the realm of LNM imaging and share our perspective on the potential trajectory of DDS development.

**Graphical Abstract:**

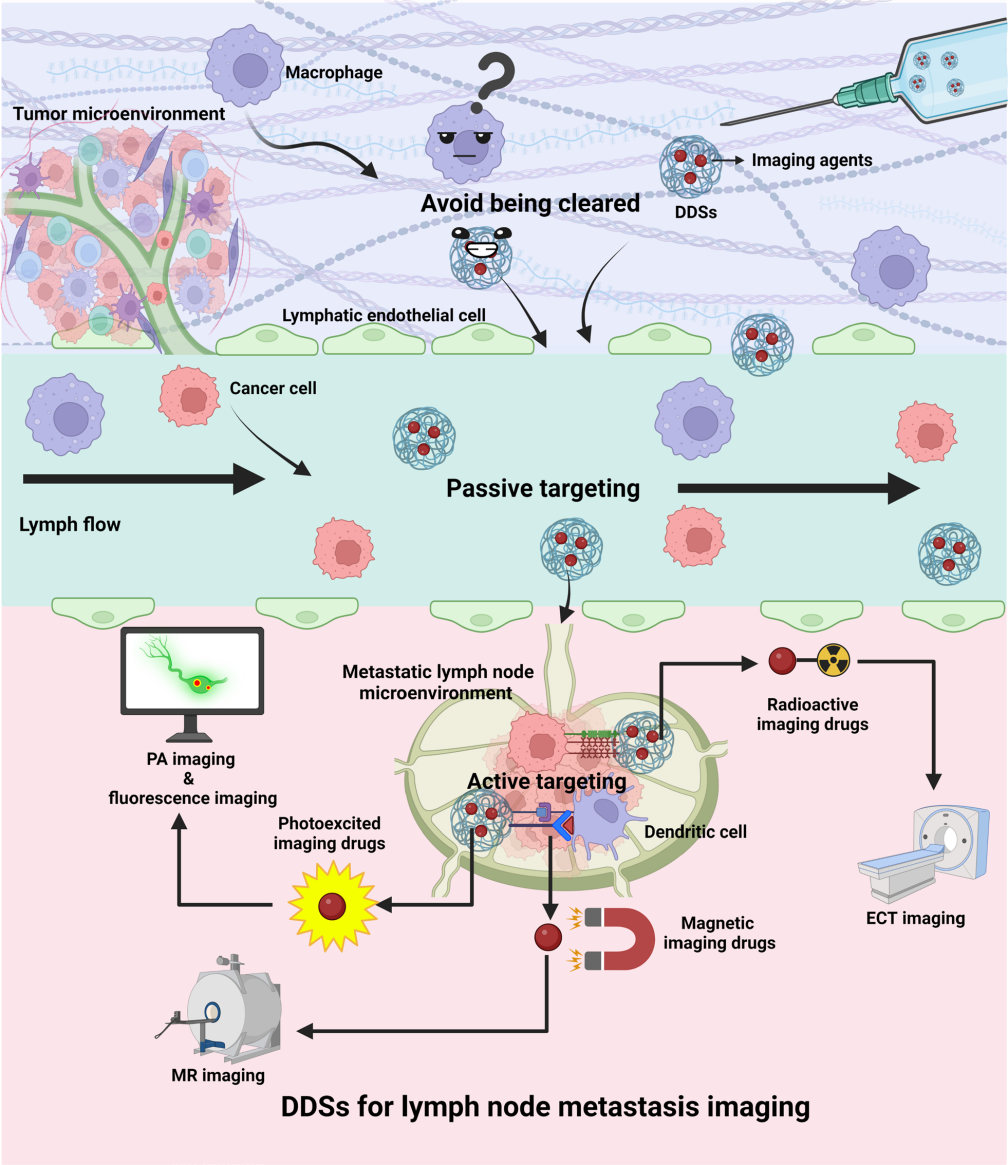

## Highlights

1. The shortcomings inherent in traditional methods of administering imaging agents and the application of drug delivery systems (DDSs) across diverse imaging techniques are elucidated.
2. The pivotal role that DDSs play in specifically targeting metastatic lymph node (LN) imaging and integrating the diagnosis and treatment of lymph node metastasis (LNM) is discussed.
3. The potential risks associated with the targeting of LNs using DDSs are detailed.
4. The prospects of utilizing DDSs in LNM imaging are explored, and insights are provided regarding the anticipated trajectory of DDSs’ future development.

## Introduction

Cancer stands as a paramount global public health concern, assuming the second rank after cardiovascular disease in terms of mortality [1]. Lymphatic invasion and subsequent metastasis to lymph nodes (LNs), leading to the propagation to other regions of the body, is a predominant characteristic of cancer cells [2]. Lymph node metastasis (LNM) significantly contributes to cancer-related mortalities and forms a key determinant in assessing the survival and prognosis of patients [3, 4]. The societal and healthcare burden attributable to cancer’s lymphatic metastasis is substantial, posing a serious threat to human health and survival.

LNs, as secondary lymphatic organs, have crucial roles in facilitating immune evasion by cancer cells and serve as primary routes for metastatic dissemination, especially in cancers such as breast, and head and neck cancer [5, 6]. LNM is indicative of a grim prognosis and functions as a relay station and booster for distant metastasis, thereby profoundly influencing survival time and life quality of patients. Moreover, LNM significantly impacts the TNM staging, guiding the treatment decision-making process which frequently involves surgical intervention for lymphatic metastasis [7].

Up to now, LN dissection remains one of the main methods for treating LNM. Although current diagnostic techniques are multifarious, imaging examination still serves as the mainstay for diagnosing LNM. The development of LN dissection evolves with the renewal of treatment concepts and the advancement of basic theories. The determination of the surgical range often depends on the imaging diagnosis; thus, defective diagnostic performance may result in a poor prognosis, such as occult LNM [8]. Furthermore, the implementation of excessive surgical procedures may also result in many adverse outcomes, such as nerve damage, pneumothorax, chyle leak, lymphedema, etc., which further lead to physiological dysfunction, disability, appearance damage, and even death [9–14]. Therefore, improving diagnostic efficiency and accurately identifying tumor LNM is critical for early cancer diagnosis and treatment, precise staging, and prognosis prediction, ultimately contributing to the reduction of missed diagnoses and treatment side effects. This improvement helps in identifying the presence of LNM, distant metastasis, and other complications. ultimately aiming to improve patient survival and quality of life [15].

**Fig. 1:**
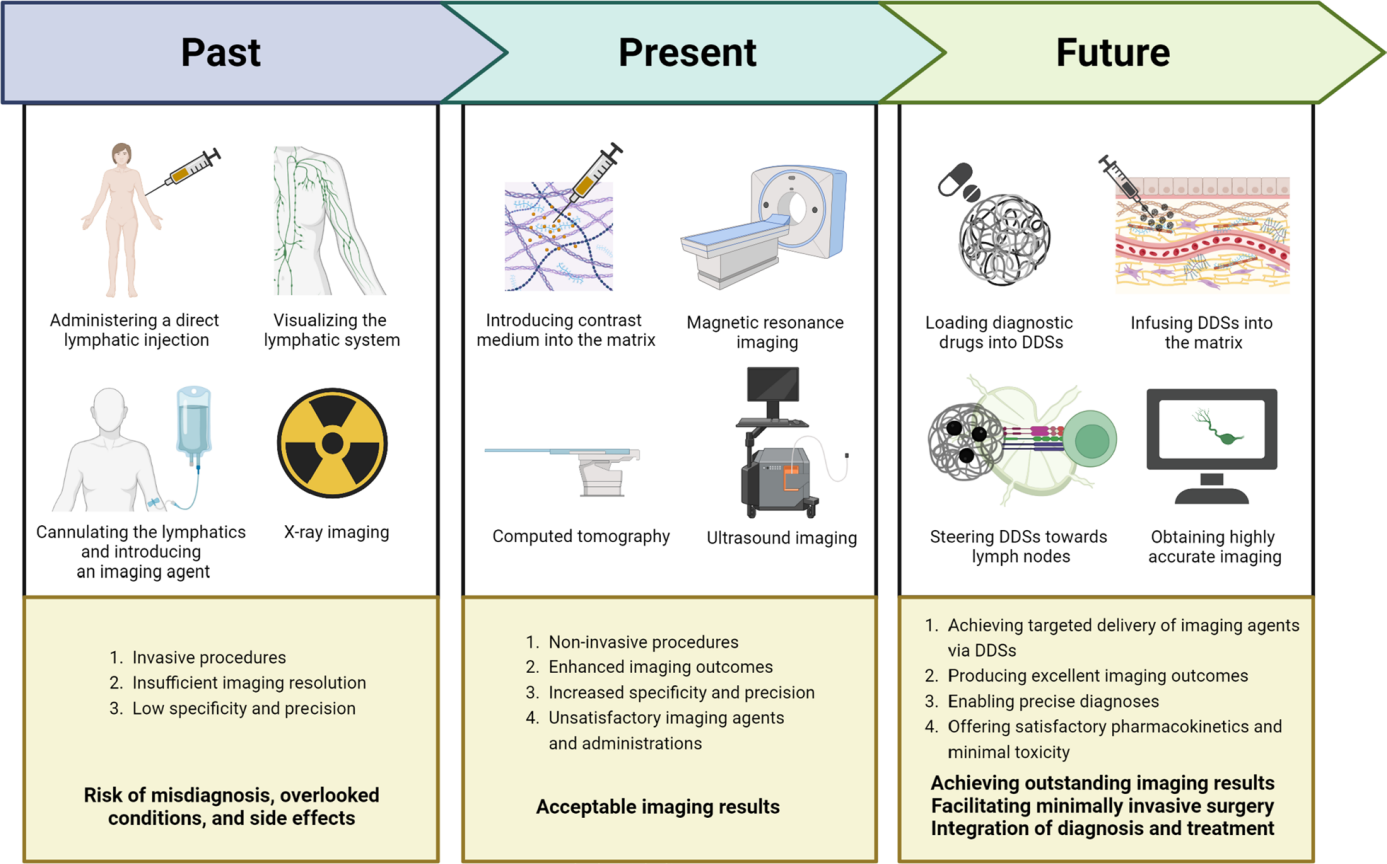
The chronological progression—past, present, and anticipated future—of lymph node (LN) imaging via the medium of drug delivery systems (DDSs). Created with BioRender.com

Medical imaging has been instrumental in diagnosing LNM. In the past, clinicians typically used X-rays to diagnose LNM. Currently, commonly used imaging techniques in the diagnosis of LNM include computed tomography (CT), magnetic resonance imaging (MRI), and ultrasound (US) imaging, among others (Fig. 1). Because the density difference between LNs and surrounding tissues is low, the introduction of contrast agents can effectively improve LNs contrast, making the image easier to identify and increasing the diagnostic performance. Contrast agents significantly enhance the visual differentiation of lymphoid tissue, bolstering diagnostic capabilities. The combination of various imaging techniques and contrast agents can achieve accurate and minimally invasive visualization of LNM [16]. However, traditional contrast agent administration faces issues such as lack of targeting, brief fluid retention time, a single imaging modality, and limited detection points; some contrast agents may also produce renal and nerve toxicity [17, 18]. It has been reported that reversible acute renal failure may occur shortly after the injection of a radioactive contrast agent, possibly due to the direct induction of renal tubular epithelial cell toxicity and renal medulla ischemia [17]. Moreover, the use of gadolinium-based contrast agents is closely related to renal systemic fibrosis and gadolinium accumulation in the brain [19]. This evidence has the potential to influence the use of contrast agents in LNM imaging. Given the limitations of traditional administration methods and the importance of accurate imaging in the diagnosis of LNM, it is urgent to propose new methods of imaging agent delivery.

Controlled drug delivery systems (DDSs) are specialized devices designed to precisely deliver drugs to targeted sites within the body. Composed of the drug (imaging agent), a targeting component, and a carrier, they form an interactive system capable of specifically delivering diagnostic agents to biological targets. Through modification, contrast agents can effectively avoid clearance by phagocytes and actively target specific sites in LNs. Some studies have shown that DDSs are highly retained in metastatic LNs due to the enhanced permeability and retention (EPR) effect. Leveraging both active and passive targeting effects, these systems offer optimized delivery of imaging agents for superior diagnostic efficacy and biosafety, facilitated by accurate drug release sites, rates, and times, compared to traditional administration methods. Some DDSs also allow fluorescence imaging techniques to image longer wavelengths beyond the traditional near-infrared (NIR) region, effectively reducing molecular scattering and interference from endogenous substances [20]. Additionally, DDSs are ideal for combining two or more contrast agents to overcome the limitations of a single imaging modality. These characteristics enable DDSs to provide accurate diagnosis and guidance for subsequent treatment.

DDSs have emerged as promising new vehicles for the delivery of imaging agents, with researchers emphasizing the pivotal role of the carrier in the DDSs’ targeting ability. Present research primarily focuses on nanomaterial carriers such as nanoparticles (NPs), and microbubbles [21, 22], that endow controlled DDSs with unique adjustability, surface effects, and size effects [23, 24]. Given the essential role of LNM localization in directing cancer staging and treatment outcomes, the amalgamation of DDSs with CT, MRI, and other imaging techniques for LNM imaging has drawn considerable attention [25–27]. DDSs exhibit properties that enable rapid clearance from the injection site, targeted transport facilitated by the targeting component [28], and evasion of the reticular endothelial system (RES) phagocytosis effect, leading to retention in LN [29, 30]. This enhances LN imaging accuracy, ensures precise metastasis localization, and reduces non-target accumulation and the dosage and toxicity of diagnostic drugs. The use of DDSs effectively mitigates the need for LN dissection, lowers postoperative recurrence rates and side effects [31], establishing DDSs as a promising avenue for metastatic LN imaging research.

In this review, we present an overview of the evolution of LN imaging, and outline the application of commonly used DDSs in conjunction with various imaging techniques for LNM imaging (Fig. 1). Furthermore, we delve into the concept and implementation of integrated diagnosis and treatment using DDSs. Lastly, we discuss the potential of DDSs in LN imaging and present our insights into the future developmental trajectory of DDSs.

## Brief history

The genesis of medical imaging can be traced back to 1895 when Röntgen captured the world’s first X-ray image of a human hand [32]. This pioneering effort marked the birth of medical imaging and catalyzed its continuous evolution, significantly contributing to disease diagnosis and treatment. In 1915, Dewis and colleagues advocated for the use of imaging tests to aid in the diagnosis of LNM when conventional diagnostic means fell short, thus averting unnecessary exploratory surgeries [33]. This initiated a novel paradigm for diagnosing subsequent LNM. By 1938, Warren et al., through their analysis of soft tissue sarcoma hospitalization cases, discovered that 5–10% of sarcomas had LNM, emphasizing the critical role of LNs in human tumor metastasis [34].

In 1971, the introduction of CT to clinical practice marked another milestone, further bolstered by Ambrose et al.’s application of CT in clinical trials [35]. This development advanced digital radiographic technology and enriched the diagnostic capabilities for ensuing diseases [36]. By 1977, Mancuso and colleagues utilized CT to appraise the association between primary lesions of laryngeal cancer and LNM, providing a foundation for subsequent CT applications in LNM diagnosis [37]. A year later, Carter successfully employed lymphoscintigraphy (LSG) to analyze LNM in breast cancer patients, obtaining satisfactory imaging results [38]. This feasibility study helped establish LSG as the gold standard for clinical lymph imaging [39].

In 1992, Husband et al. integrated MRI into the imaging of LNM in bladder cancer, yielding enhanced contrast between LNs and blood vessels as compared to prior imaging techniques [40]. In 2004, indocyanine green (ICG), the first cyanine dye approved by the US Food and Drug Administration (FDA), was utilized for sentinel lymph node (SLN) detection in gastric cancer patients in combination with the NIR lymphography technique [41], affirming the utility of NIR lymphography in diagnosing LNM.

The evolution of DDSs was catalyzed in 1952 by the development of the first controlled release preparation by Smith and colleagues [42]. Subsequently, in 1976, Kreuter introduced the concept of “nanoparticle” in medicine [43]. As effective carriers and imaging drugs, NPs have progressively found their place in DDSs. In 1984, Nefzger and colleagues discovered that NPs exhibited high retention in tissues such as LNs, liver, stomach, and bone marrow post-administration, foreshadowing the successful application of DDSs in LNM imaging [44].

In 1989, Pouliquen and colleagues engineered a superparamagnetic NP to function as a contrast agent for liver MRI, effectively amplifying hepatic contrast and thus promoting the application of DDSs in imaging [45]. In 1994, Anzai et al. pioneered the application of DDSs in imaging LNM in head and neck cancer patients, observing that benign LNs exhibited significantly lower signal intensity than metastatic LNs post-injection, aiding in their differentiation and accurate diagnosis [46]. In 2004, Kobayashi and colleagues employed DDSs for lymph imaging by MRI to visualize the drainage from mouse mammary tumors to lymphatic vessels and LNs. The lymphatic drainage visualization capability of DDSs outperformed that of conventional administration methods, providing a clearer depiction of lymphatic drainage [47].

**Fig. 2:**
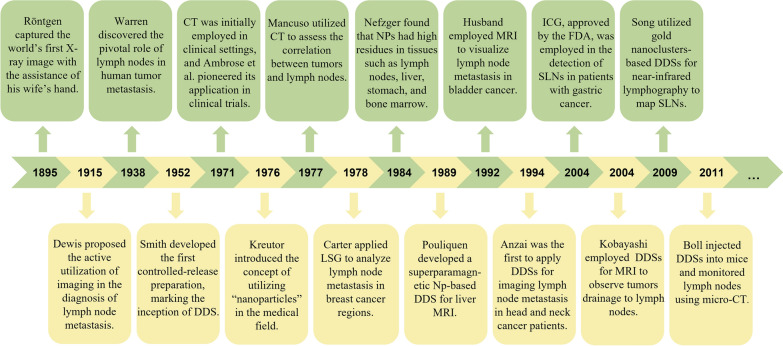
A brief historical overview of lymph node metastasis imaging based on DDSs. *DDS* drug delivery system, *CT* computed tomography, *LSG* lymphoscintigraphy, *NP* nanoparticle, *MRI* magnetic resonance imaging, *ICG* indocyanine green, *SLN* sentinel lymph node

In 2009, Song and colleagues employed gold nanoclusters-based DDSs as imaging agents for NIR lymphography to map SLNs [48]. Subsequently, in 2011, Boll and colleagues injected NP-based DDSs into mice and found that DDSs provided strong contrast for abdominal, mediastinal LNs, and adrenal glands with a low dose requirement when monitoring liver diseases using micro-CT [49]. These advances highlight the potential of DDSs in precise imaging and differential diagnosis of tumor LNM, warranting further research and clinical applications (Fig. 2).

## Cancer-induced alterations in LNs

The lymphatic system, which includes a multitude of organs such as the bone marrow, spleen, tonsils, thymus, and LNs, interconnected by lymphatic vessels, serves as the body’s second vascular system. It plays crucial roles in maintaining fluid homeostasis and regulating adaptive immune responses [50]. The ensuing discussion explores LN modifications induced by cancer cell infiltration.

### Imaging-based detection of metastatic alterations in LNs

Tumor metastasis to LNs causes changes in the size, shape, and structure of LNs, which can be detected by imaging examination to confirm the occurrence of LNM [51]. These modifications include cancer cell infiltration into LNs, eliciting inflammatory responses and altering the nodes’ size and shape.

Apart from external morphological shifts, metastatic LNs may also undergo structural modifications. The edges of metastatic LNs are usually sharper than those of benign LNs, a characteristic related to tumor cell infiltration in LNs [52]. This is because the infiltration of normal LN tissue by cancer cells can enhance the acoustic impedance within the LN, making the boundaries more distinctive in imaging compared to normal LNs. Invasion by cancer cells can also compromise or obliterate the fatty tissue in the hilum of the LN, leading to the loss of a low-density structure on imaging [53, 54]. However, a blurry border of a confirmed metastatic LN may indicate extracapsular infiltration by cancer cells, which usually indicates a worse prognostic outcome [55]. Additionally, metastatic LNs may exhibit features such as centrifugal cortical hypertrophy [56], intranodal necrosis [57], calcification, and the corresponding characteristics of the intranodal echogenic hilus, cystic area, or a punctate acoustic shadow, among other imaging features [58]. By identifying these alterations, imaging techniques can assist in diagnosing LNM.

In the past, there was no clear consensus on the diagnostic criteria for imaging LNM. The diagnosis of LN imaging still relied mainly on the experience of physicians. The introduction of the Node Reporting and Data System (Node-RADS) addressed this problem and facilitated the standardization of imaging evaluation of affected LNs [59]. Evaluation according to the size, configuration, boundary, and other categories within this system can effectively assess the involvement of LNs, providing a structured and repeatable criterion for the diagnosis of LNM to address the consensus and experience gap between radiologists. At the same time, it also reduces the risk of missed diagnosis due to varying diagnostic criteria (such as the missed diagnosis of micro-metastatic LNs) [60]. Currently, this system has been applied to the LN imaging of patients with bladder cancer, colon cancer, cholangiocarcinoma, lung cancer, etc. Compared with traditional non-standardized diagnostic criteria, the overall diagnostic performance of the new system has improved [60–63]. Nevertheless, these shape and structure changes should not be used as standalone diagnostic markers, and a definitive diagnosis requires a combination of multiple indicators and pathological examination results.

### Physiological basis of DDS in LN imaging

Lymphatic capillaries, positioned at the terminus of the lymphatic system, gather lymphatic fluid from interstitial tissues and transport it back to the cardiovascular system via lymphatic vessels, thereby maintaining the fluid balance of the circulatory system. The broad intercellular space and high permeability of the endothelial cells in lymphatic capillaries facilitate the passage of interstitial fluid, which is subsequently collected as lymphatic fluid [16]. Capitalizing on this characteristic, DDSs with a diameter less than 200 nm can enter the lymphatic vessels and flow into the LNs [64].

**Fig. 3:**
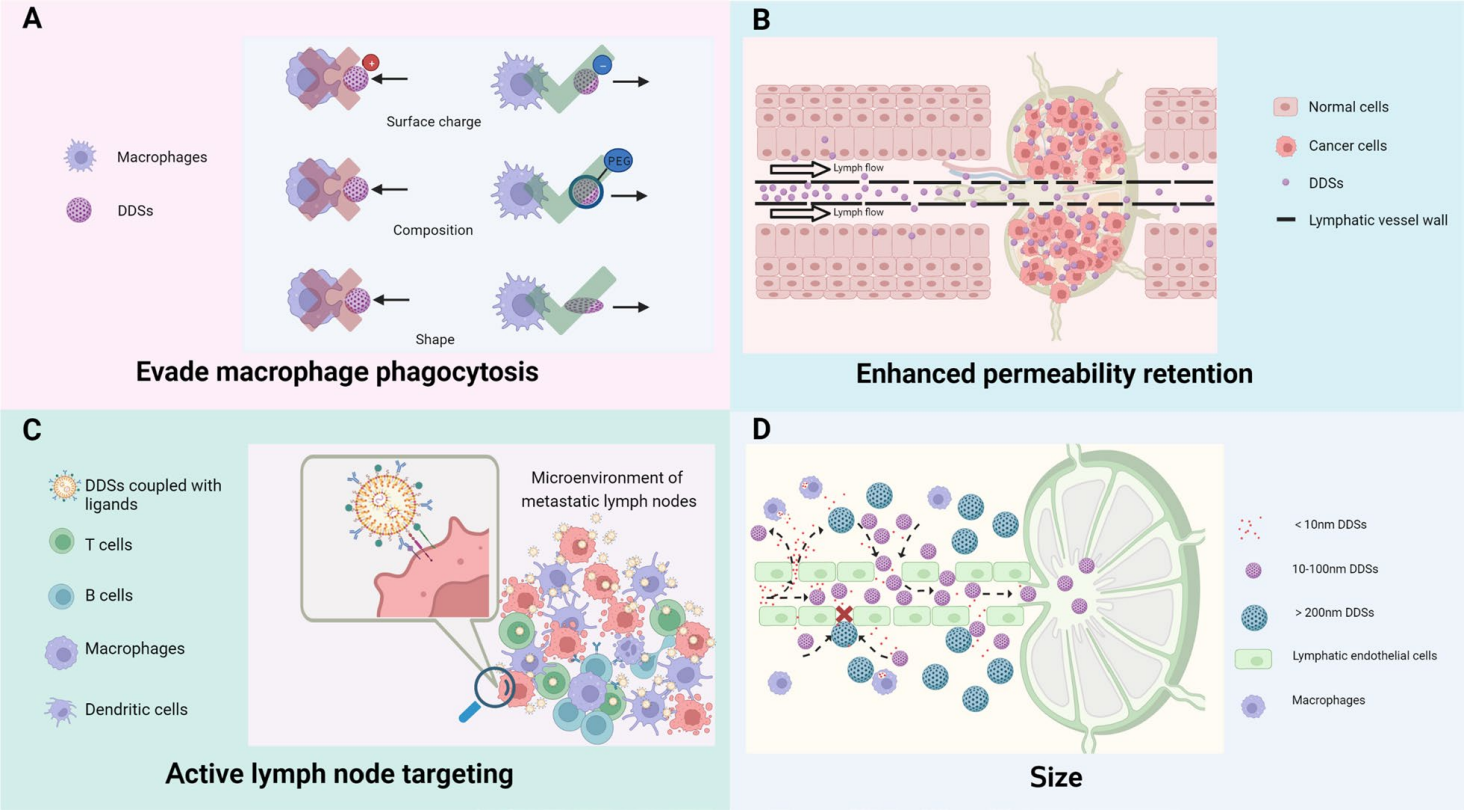
The targeted lymph node mechanisms of drug delivery systems (DDSs). **A** By modifying their surface charge, composition, and shape, DDSs possess the capability to elude phagocytosis activity by macrophages. **B** Benefitting from the EPR effect, DDSs can maintain a persistent presence within metastatic lymph nodes. **C** Leveraging the use of ligand-coupled NPs, DDSs have the potential to actively home in on targeted lymph nodes. **D** DDSs, when tailored to a specific size, gain the ability to infiltrate lymphatic vessels in substantial volumes. Created with BioRender.com

During lymphatic return, macrophages remove foreign material. To evade macrophage clearance and amplify their passive targeting ability towards LNs, DDSs undergo specific modifications in shape, surface charge, and composition, effectively impeding their clearance by macrophages. In terms of shape modification, non-spherical NPs can effectively reduce the likelihood of clearance by phagocytes, whereas spherical NPs are more susceptible to blood convection and tend to drift laterally along the walls of blood or lymphatic vessels, thus reducing the likelihood of margination [65, 66]. Regarding surface charge, negatively charged DDSs enhance the efficiency of transport to LNs and effectively promote the activation of immune cells in the tumor microenvironment of metastatic LNs. They can be preferentially taken up by cancerous tissues and reduce the non-specific clearance of macrophages [67]. Concerning composition, the composition of DDSs affects the pharmacokinetics of their internal drugs and the immune system’s clearance efficiency. For example, PEGylation—a technique that modifies compounds or supports by adding polymeric chains of ethylene glycol (or polyethylene oxide, or polyoxyethylene)—is a widely used DDS structure modification strategy. It endows DDSs with inertness and stability, mitigates the interaction between the drugs and the biological milieu, confers stealth effects on DDSs, diminishes protein adsorption, and prevents cellular ingestion (Fig. 3A) [68–71]. It has been demonstrated that, compared to healthy LNs, metastatic LNs exhibit increased lymphatic vessel permeability and obstructed lymphatic drainage, which facilitate the retention of DDSs and augment their passive targeting effect through the EPR effect (Fig. 3B) [72].

LNs are known to house a profusion of immune cells such as dendritic cells (DCs), T cells, B cells, among others. These cells sample the incoming lymphatic fluid to capture antigens and initiate adaptive immune responses [73]. Upon entry into LNs, appropriately sized DDSs can be engulfed by antigen-presenting cells (APCs), thereby serving a passive targeting role (Fig. 3D). Furthermore, APCs in LNs express mannose receptors [74], CD11c [75], CD169, and other specific targets [76]. The cancer cell infiltration in metastatic LNs also exhibits abnormally high expression of molecules like CD44 [77]. Conjugation of DDSs to ligands for these targets can enable active targeted delivery to metastatic LNs (Fig. 3C). Recognizing the imaging potential of DDSs, researchers have committed themselves to the development of LNM imaging using DDSs. This aims to enhance the efficiency and precision of diagnosing cancer LNM through the introduction of this delivery system (Fig. 3).

## Current techniques in LN imaging

Several methods for lymphatic imaging are available, enabling visualization of LN structures in various body parts and diagnosing LNM. The minimal density difference between LNs and surrounding tissues, however, often complicates the diagnosis of metastasis through imaging, leading to misdiagnoses and missed diagnoses [78]. Frequently, contrast agents are employed to enhance the imaging effect and improve tissues contrast. Although many articles detail non-targeted and traditional LN imaging techniques, this section focuses on the principles and limitations of imaging agents within these traditional administration methods [79]. This coverage encompasses numerous imaging techniques employed in medical diagnostics, such as X-ray scans and digital imaging methods.

Digital imaging techniques, such as CT, MRI, US imaging, NIR lymphography, and other LN imaging techniques, have revolutionized the medical field by enabling high contrast, high resolution, and minimally invasive LN imaging [80–82]. Despite these advancements, traditional administration methods paired with various imaging techniques, while offering acceptable results, continue to face issues such as lack of targeting, brief fluid retention time, and limited detection points. These drawbacks inhibit the effectiveness of diagnosing LNM [83, 84].

### X-ray imaging

The earliest approach to LN imaging involved the use of X-rays to visualize LNs post the injection of contrast agents [85]. However, this method necessitates invasive contrast agent injections, conflicting with modern non-invasive diagnostic philosophies and negatively impacting patient compliance [86].

### LSG and SPECT/CT

LSG, recognized as the gold standard for LN imaging, is routinely employed to assess lymph transport and identify metastatic LNs associated with cancer [39]. This procedure encompasses the administration of a tracer, imbued with a radioactive isotope, into interstitial tissues. Subsequently, a single-photon-emission-computed-tomography (SPECT) instrument external to the body is utilized to monitor the tracer’s path.

Coupling LSG with SPECT/CT facilitates three-dimensional imaging of the lymphatic system, thereby accurately locating metastatic LNs [87, 88]. This combined approach, when utilized with DDSs, proves instrumental for the precise localization of the SLN and for effectively guiding LN biopsy in breast cancer patients [89].

Dilege et al.’s study indicated that this imaging methodology was able to accurately identify all sampled LNs. Notably, 88.5% of the patients’ clipped nodes were classified as SLN in this study. This enables early detection and precise excision of metastatic LNs, thus mitigating the likelihood of postoperative complications [90].

However, there exist limitations with non-NP-based Technetium-99 (99mTc) complexes, commonly employed in this context. Shortcomings such as a brief half-life and suboptimal contrast degrade the diagnostic value of the resultant imaging.

### PET/CT

Glucose serves as the principal energy source for human cells. Exploiting the Warburg effect, cancer cells hinder the tricarboxylic acid cycle and preferentially use the glycolytic pathway for energy provision, albeit with lower efficiency. Consequently, malignant tumors display a markedly higher uptake of glucose compared to normal tissues [91].

Positron-emission-tomography (PET)/CT leverages this distinct trait of malignant tumors by employing radiolabeled glucose analogs as tracers to image pathological tissue. Herbrik et al., for instance, utilized 18F-fluorodeoxyglucose (18F-FDG) as a tracer to investigate LNM in patients diagnosed with non-small cell lung cancer, achieving an accuracy rate of 81% [92]. Similarly, Billé et al. employed 18-FDG to detect LNM in a cohort of 159 non-small cell lung cancer patients, documenting a specificity of 91.9% and an accuracy rate of 80.5% [93].

Nevertheless, FDG-PET/CT has exhibited limited sensitivity in detecting LNM in cases of esophageal squamous cell carcinoma, with a range of merely 30–40%. This could potentially result in false negative diagnoses [94, 95]. Furthermore, certain diseases may confound the diagnosis of metastatic LNs via FDG-PET. For instance, nodular lymphoid hyperplasia of the lung, a benign, non-neoplastic lesion, can manifest as multifocal lesions bilaterally, and its FDG-PET results can be falsely positive, potentially leading to misdiagnosis [96, 97]. Additionally, Manta et al. performed an FDG-PET/CT scan on a patient suspected of having thyroid malignancy. The results revealed a strong FDG uptake in the mediastinal and bilateral hilar LNs. However, post-surgical excision, the pathological examination revealed non-necrotizing granulomatous lesions, indicative of thyroid nodule disease [98]. This misdiagnosis could potentially prompt incorrect treatment strategies, thereby inflicting undue trauma on the patient.

### MRI

MRI operates by inducing alignment of hydrogen atoms within the body using a magnetic field. The subsequent emission of radio waves following disturbance is captured and processed by an advanced computer system, culminating in the generation of detailed images depicting diverse tissues and other critical anatomical structures [99, 100].

Gadolinium-based contrast agents (GBCAs), such as Gd-DOTA, Gd-DTPA, are presently employed extensively for contrast-enhanced MRI. Owing to its strong paramagnetism, Gadolinium (Gd) influences tissue contrast by stimulating the relaxation of nearby hydrogen protons, facilitating indirect imaging [101, 102]. A study demonstrated that post-injection of Gd-DTPA, MRI yielded a sensitivity of 91.1% and accuracy of 87.2% in detecting metastatic LNs in patients with nasopharyngeal carcinoma. Precise and efficient localization of the SLN substantially mitigates unnecessary tissue resection, lessens surgical side effects, and enhances patient quality of life [103].

However, GBCAs are associated with certain drawbacks. Firstly, GBCAs exhibit potential cytotoxicity. Research has revealed that this contrast agent incurs nephrotoxicity in patients with chronic kidney disease, potentially resulting in nephrogenic systemic fibrosis. Secondly, the excretion of GBCAs poses a challenge. Even in patients with a normal glomerular filtration rate, the use of GBCAs can lead to gadolinium deposition in multiple organs. Certain patients may manifest symptoms such as skin burning pain, muscle cramps, and ‘brain fog’, indicative of gadolinium deposition disease [104–106]. As Gd can persist in the brain for prolonged periods, the FDA has issued a warning about “GBCA retention in the body” [107], and the European Medicines Agency (EMA) has classified GBCAs as a high-risk imaging agent [108].

### NIR fluorescence imaging

NIR fluorescence imaging technology, following the administration of a fluorescent dye, stimulates the fluorescent properties of this dye through a detector, thus facilitating visualization the contrast agent and enabling LN imaging. Within the NIR spectrum, human tissue does not exhibit autofluorescence, thus yielding high contrast favorable for imaging and observation [109]. Additionally, given that NIR light is invisible, the use of this fluorescent dye minimizes visual impact on patients, thereby reducing potential negative emotional responses [110].

ICG, a contrast agent approved by the FDA for clinical use [41], boasts robust tissue penetration capacity attributable to its excited fluorescence (compared to traditional blue dyes, which are easily obscured by dense tissues like fat, leading to inadequate imaging depth [111]), superior biocompatibility, and non-radioactive properties [112]. ICG has been broadly utilized in NIR fluorescence imaging [113, 114], offering real-time and accurate detection of metastatic LNs [115–117].

But there are certain drawbacks associated with ICG that compromise the efficacy of LNM imaging. Firstly, ICG’s amphiphilic properties and poor stability in aqueous solutions lead to aggregate and self-quenching in body fluids, thereby inhibiting the ability of imaging agents to reach the LNs [118]. Secondly, ICG’s low molecular weight hampers its retention in the SLN, resulting in potential drainage to other LNs or clearance via blood vessels, thus reducing imaging specificity [119]. Moreover, the first NIR window (NIR-I) (700–900 nm) is impacted by signal scattering of biological endogenous substances, resulting in excessive background signal in the image and inadequate tissue contrast, which compromises image-based diagnosis [120, 121].

Additionally, the toxicity of ICG correlates with light duration [122], indicating that the effectiveness of LNM detection using ICG hinges on the physician’s expertise and technical acuity. This could potentially lead to a disparate distribution of medical resources and unequal access to medical diagnosis and treatment for patients. Therefore, the redefinition of LNM diagnostic principles and strategies is pivotal, not only to enhance survival and quality of life for cancer patients but also to promote social equity.

### US imaging

US imaging operates on the principle of sound wave reflection. It gathers these reflected sound waves from tissue organs and converts them into images [123]. Some researchers have attempted to augment the contrast of US imaging using microbubbles (size < 10 μm) encapsulating diagnostic gas, thereby procuring high spatial–temporal resolution images. However, their constrained contrast and indistinct tissue boundaries may compromise the diagnostic accuracy for LNM [22, 124].

## DDSs for targeting and imaging of LNs

In recent decades, the use of DDSs has expanded considerably. These systems find widespread application in drug delivery, tissue engineering, and medical imaging, among other fields [125–127]. Particularly within the realm of LN imaging, scholars have begun investigating DDSs due to their characteristics of reducing toxicity and enhancing action, aiming to overcome the limitations of traditional imaging methods and agents. A large number of studies have demonstrated the effectiveness of DDSs in LNM imaging [128–131]. Specifically, DDSs based on NPs possess unique characteristics of a high surface area-to-volume ratio, which can achieve strong and longitudinally stable imaging signals. By reducing the unexpected reaction between the drug and the body’s microenvironment, controlling drug release, and altering biological distribution, the toxic and side effects of the contrast agent on the body can be minimized. Secondly, the strategy of active and passive targeted delivery of DDSs can detect the desired target and improve the sensitivity and specificity of imaging. Additionally, DDSs can protect the drug from degradation in the body, improve the drug’s bioavailability, and ultimately achieve the purpose of reducing toxicity and increasing efficiency [132]. Various forms of DDSs, such as wafers, foams, films, hydrogels, NPs, and fibers, have been developed [133–138]. Among them, NPs are the most popular in the realm of LN imaging and can be roughly classified into lipid NPs, radioactive nano-colloids, metal NPs, magnetic NPs, etc. [139–142]. Available preparation methods include self-assembling systems, microfluidic production, aqueous coprecipitation, thermal decomposition, sol–gel reaction, etc. [132, 143, 144].

Depending on the disease, the desired effect, and the characteristics of the drug, the route of administration of DDSs can usually be divided into oral, parenteral, transdermal, and nasal administration, among others [145]. Parenteral administration is currently the most commonly used invasive route of DDS administration, with advantages including bypassing first-pass metabolism, rapid onset, controllable drug utilization, reduction of gastrointestinal irritation, and reliability for critically ill patients. Parenteral administration can further be divided into subcutaneous, intramuscular, and intravenous injection, with the absorption and onset rate of the drug increasing respectively [146]. At present, DDSs used in LNM imaging can be roughly divided into two methods: one is to increase the efficiency of delivery (e.g., 99mTc-labeled colloids), and the other is to increase the specificity of delivery (such as surface modification with tumor antigen ligands). This paper will categorize various imaging techniques that utilize DDSs and present them in tabular form (Table 1). In the following sections, we will review the delivery system represented by NPs.

**Table 1:** Imaging technologies based on drug delivery systems (DDSs)

Imaging technologies	Principle	Example of DDSs	Advantages	Refs.
LSG and SPECT/CT	Injecting a tracer containing a radioactive isotope into the interstitial tissues and using external detectioninstr-ument (SPECT) to track and image it	99mTc-labeled nanocolloid DDSs (99mTc-sulphur colloid, 99mTc-nanocolloidal albumin, 99mTc antimony trisulfide colloid, 99mTc-etarfolatide, etc.)	The short half-life and poor contrast of the 99mTc complex are improved; Helping in the early detection of SLN; Guiding lymph node biopsy effectively; Reducing the occurrence of postoperative sequelae, etc	[147, 149–151]
PET/CT	Tumor tissues exhibit abnormally increased glucose uptake. PET/CT utilizes radiolabeled glucose analogs as tracers to image the lesion tissue based on this characteristic of malignant tumors	68 Ga-labeled targeted PSMA-DDS; 124I-labeled antibody against LYVE-1 DDS	Increasing the sensitivity and accuracy of diagnosis and reducing the occurrence of misdiagnosis and missed diagnosis; Improving the targeting of imaging agents to lymph nodes, etc	[167, 168]
MRI	MRI uses strong magnetic fields and radio waves to create detailed images of the body's internal structures. The magnetic field aligns the body's protons, and the radio waves cause them to emit signals which are detected and analyzed to create images	Gd_2_O_3_PCD coated DDS; USPIO DDSs	Reducing Gd deposition and cytotoxicity; Pharmacokinetics and biocompatibility are satisfactory; Having higher diagnostic specificity and sensitivity, etc	[171, 172, 174]
NIR fluorescence imaging	NIR fluorescence imaging technology is used to visualize lymph nodes. It involves injecting a fluorescent dye and exciting its fluorescent properties using a detector to create contrast agent visualization	PLGA-ICG DDSs; γ-PGA-ICG DDSs; PEG coated ICG-DDSs; Metal nanoclusters DDSs	Good retention rate and stability in vivo; Having a stronger NIR fluorescence signal than ICG; Increasing the molecular weight to reduce clearance by blood; Serviceable tissue penetration and lower interference signals from endogenous substances, etc	[111, 180, 188, 190]
US/PA imaging	US imaging is based on the principle of sound wave reflection, collecting the reflected sound wave from the tissue organs and converting them into images. PA imaging uses laser-generated light pulses to generate acoustic waves in the body's tissues which are detected and analyzed to create images	Carbon NP DDS; Cu-neodecanoate DDS	Satisfactory SLN imaging capability, imaging depth, signal noise, ratio and tissue contrast, etc	[194–196]
Multimodality imaging	Combining different lymph node imaging technologies	GC-AuNCs/ICG DDS (PA/Fluorescence imaging); Nanoprobes DDSs (PA/Fluorescence imaging); Iron oxide-ATF DDS (NIR/PA/MR I)	To compensate for the limitations of different technologies, take advantage of each technology. Explore the potential of lymph node imaging technology based on DDSs	[198–201]

*LSG* lymphoscintigraphy, *SPECT* single-photon-emission-computed-tomography, *CT* computed tomography, *DDSs* drug delivery systems, *SLN* sentinel lymph node, *PET* positron-emission-tomography, *PSMA* prostate-specific membrane antigen, *LYVE-1* lymphatic vessel endothelial hyaluronan receptor-1, *MRI* magnetic resonance imaging, *USPIO* ultrasmall superparamagnetic particles of iron oxide, *NIR* near-infrared, *PLGA* poly (DL-lactic-co-glycolic acid), *ICG* indocyanine green, *PEG* polyethylene glycol, *US* ultrasound, *PA* photoacoustic, *NP* nanoparticle, *ATF* amino-terminal fragments

### LSG and SPECT/CT

In LSG, the controlled administration strategy most frequently utilized involves NP-colloid-based DDSs labeled with the radioactive isotope 99mTc. This approach mitigates concerns associated with short half-lives and suboptimal contrast. An example is 99mTc-etarfolatide, a DDS carrying a 99mTc-labeled folate conjugate, designed for imaging targeting folate receptors on tumor cells. Preclinical studies have shown that 99mTc-etarfolatide has a higher affinity with human folate receptors, thus improving the specificity of imaging [147, 148]. Drawing on the inherent spatial characteristics of NP carriers [24], a diverse range of 99mTc-labeled DDSs are currently in use, distinguished by the differing particle sizes of their NP carriers. For instance, 99mTc-sulfur colloid (particle size exceeding 100 nm) [149], 99mTc-nanocolloidal albumin (particle size less than 80 nm) [39, 150], and 99mTc antimony trisulfide colloid (particle size between 3 and 30 nm) [151].

The capacity for passive targeting of LNs by DDSs is determined by a variety of factors [152, 153], of which the particle size of NP carriers emerges as the most significant in LSG LN imaging [154, 155]. Particle sizes of less than 10 nm expedite clearance of DDSs from the interstitial fluid and allow for entry into the LN via the lymphatic vessel wall. However, such small particles can also easily traverse capillaries and thus be cleared prematurely. Conversely, DDSs with particle sizes greater than 200 nm are more likely to remain entrapped in the interstitium and be eliminated by the RES. The optimal particle size for DDSs, therefore, typically ranges from 10 to 100 nm. This range allows for broad and effective aggregation within metastatic LNs [155–158]. However, no consensus has been reached regarding the most suitable particle size range for DDSs [159, 160].

DDSs with an appropriately sized particle can achieve significant regional LN retention. This facilitates intraoperative positioning via a γ probe and allows for accurate and repeatable SLN localization [161, 162]. This precision reduces the need for radical LN dissection surgery. It is crucial to remember, however, that radioactive isotopes possess inherent decay properties. Therefore, the attenuation correction of results is indispensable to ensure accuracy [163].

### PET/CT

The conventional radiolabeled tracers, epitomized by 18F-FDG, although possessing high sensitivity and specificity in detecting tumor metastasis, present significant limitations, consequently lowering diagnostic accuracy and restricting their diagnostic value. Due to the non-targeted nature of traditional contrast agents, the potential toxicological side effects of imaging agents during the diagnostic process are particularly prominent. Moreover, the non-targeted feature restricts the accuracy of diagnosis and the ability to distinguish small lesions. These shortcomings underscore the need for an altered administration approach, one that promises enhanced sensitivity and precision.

Recent studies have begun to employ surface-modified DDS tracers for LN imaging, a technique that facilitates the entry of DDSs into LNs and enhances the targeting of imaging agents [164–166]. Schilham et al. utilized tracers based on 68 Ga-labeled targeted prostate-specific membrane antigen (PSMA) DDS, injecting these into patients with prostate cancer to detect LNM. The modified PSMA DDS demonstrated superior sensitivity to LNM compared to 18F-FDG, with diagnostic accuracy reaching up to 85% [167].

In another study, Mumprecht administered a 124I-labeled antibody against the lymphatic vessel endothelial hyaluronan receptor-1 (LYVE-1) DDS to mice carrying the B16-F10-luc2-VEGF-C tumor with LNM [168]. These surface-modified, immunotargeted DDSs showed heightened sensitive to LNM compared to 18F-FDG [169], accumulating in high concentrations at specific sites within LNs. This undoubtedly yielded significant improvements in the diagnostic accuracy of PET-CT [170].

### MRI

Although MRI plays a significant role in displaying soft tissue imaging, the side effects of GBCAs might not be acceptable to clinical doctors and patients. The deposition of GBCAs in the brain could lead to symptoms related to central nervous system toxicity or neuroinflammation, which has been a particular concern for the EMA [103, 106]. To address the aforementioned challenges associated with GBCAs, several studies have successfully incorporated DDSs into magnetic resonance LN imaging, yielding substantial improvements. One such strategy involved modifying Gd with polycyclodextrin (PCD) to generate a Gd_2_O_3_PCD-coated DDS. When applied to a mouse model of breast cancer, this system demonstrated the capability to deliver GBCAs at lower dosage requirements, thereby enhancing imaging localization of LNM. Thanks to the Gd-modified coating, which effectively reduced Gd leakage and required lower concentrations, this DDS can decrease Gd deposition and cytotoxicity. This reduction mitigates the side effects associated with detection and enhances the biosafety of imaging [171]. Further to this, ultrasmall superparamagnetic particles of iron oxide (USPIO) currently represent the most extensively studied and utilized NPs for MRI [172–175]. Ferumoxtran-10, a type of USPIO, presents wide-ranging application prospects [176]. This compound is absorbed by macrophages, targeted, and transported to LNs where it is primarily distributed [177]. With satisfactory pharmacokinetics and biocompatibility, it proves suitable for MRI imaging detection of human LNs [178, 179]. Importantly, the use of USPIO-based DDSs for LNM detection demonstrates higher diagnostic specificity and sensitivity compared to MRI detection techniques not based on DDSs. Koh DM et al.’s research showed that, compared with non-DDS-based imaging diagnosis, LNM detection using DDSs based on USPIO shows higher diagnostic specificity, with an average specificity increase from 75 to 93% [175].

### NIR fluorescence imaging

ICG forms aggregates in body fluids and lacks stability in aqueous solutions, which hinders its effective delivery to LNs. Its low molecular weight results in poor retention in SLNs, thus reducing imaging specificity. Moreover, the NIR-I window also suffers from signal scattering and excessive background noise, which can undermine diagnostic accuracy [119]. To surmount the constraints of ICG in NIR LN imaging, researchers have sought to employ DDSs to enhance ICG delivery. DDSs, such as ICG encapsulated with poly (D,L-lactic-co-glycolic acid) (PLGA), showed a release of 78% of the encapsulated ICG within the initial 8 h of bodily introduction, with the residual portion being discharged in the subsequent 16 h. Compared to its unencapsulated counterpart, ICG-NaI, PLGA-ICG demonstrated an eight-fold increase in retention rate [180].

Poly (γ-glutamic acid) (γ-PGA)-ICG DDSs also showcase promising features. They display notable stability in an aqueous solution, resist aggregation and self-quenching at physiological temperatures, and produce a more robust NIR fluorescence signal than conventional ICG. The augmentation of their molecular weight aids in diminishing blood clearance, thereby enhancing their targeted delivery capacity to LNs and improving the imaging capabilities for LNM [111].

Modifying ICG DDSs with polyethylene glycol (PEG) polymers may also mitigate the adverse effects of ICG on LN contraction and dilatation [111, 181]. In addition to advancements in imaging materials for the NIR-I window, continuous efforts are underway to develop materials for the second NIR window (NIR-II) (1000–1700 nm). This development aims to address the shortcomings of NIR-I imaging, focusing on achieving superior tissue penetration with less signal interference from endogenous substances and improving imaging contrast [182, 183].

In this regard, metal nanoclusters have piqued researchers’ interest due to their unique attributes. As nanomaterials with a particle size ranging 1–40 nm, they exhibit distinctive surface plasmon resonance (SPR) characteristics with high adjustability [184, 185]. Notably, gold is extensively utilized in the development of nanocluster-based DDSs due to its excellent biocompatibility, size adjustability, and surface treatment capabilities [186].

By altering the structure and composition of gold nanocluster-based DDSs, the SPR position can be modified. If the SPR peak is situated in the 700–900 nm region, it can serve as a NIR-I fluorescence imaging contrast agent [187]. Conversely, when the SRP peak of gold nanocluster-based DDSs is set at 1000–1700 nm, these DDSs function as a NIR-II imaging agent, whose long-wavelength, low-scattering properties permit photons to penetrate deep tissue [188]. Research has revealed that the imaging depth of NIR-II imaging using gold nanoclusters can extend to 6.1 mm subcutaneously, a marked improvement from the 5 mm depth achieved by ICG [189]. This approach also offers high stability, sensitivity, and superior imaging resolution [190, 191].

Further enhancements to gold nanoclusters were made by incorporating targeting molecules, thus endowing the gold nanoclusters with specificity for SLN imaging. These gold nanoclusters were then modified with sulfhydryl ligands targeting DCs. Following injection of this DDS into mice, a significant accumulation in metastatic LNs was observed. This greatly enhanced imaging specificity and markedly improved the ability to identify LNM [192].

### US/PA imaging

Efforts have been made by researchers to enhance the efficacy of acoustic imaging through the exploitation of the photoacoustic (PA) effect. When human tissue absorbs and subsequently releases pulsed lasers, this expansion and contraction process generates US waves. Since different tissues absorb light to variable extents, this results in the production of US waves with differing intensities [193].

In integrating the properties of PA and US, researchers have achieved PA/US dual-mode imaging, utilizing optical excitation and acoustic detection. This approach leverages the unique attributes of the nanomaterial carriers used DDSs [31]. For instance, the specific spatial size effect of carbon NP-based DDSs allows for facilitated entry into the lymphatic system [23]. While macrophages in healthy LNs can clear these DDSs, metastatic LNs, infiltrated by cancer cells, exhibit a significant decrease in macrophages, which results in reduced DDS uptake.

Owing to these properties, carbon NP-enhanced PA/US LN imaging offers superior SLN imaging capability, along with improved PA/US imaging depth, tissue contrast, and signal noise ratio. This technique thus demonstrates higher recognition and diagnostic capabilities for LNM compared to conventional US imaging [194]. Furthermore, carbon nanomaterial-enhanced PA imaging can achieve a greater penetration than NIR imaging based on ICG, reaching an imaging depth of over 12 mm [195].

Dipanjan et al. proposed an advanced strategy in which they mixed polystyrene and Cu-neodecanoate complexes, coating the resultant mixture with phospholipids to enhance imaging. This DDS exhibits a broad light absorption spectrum and provides exceptional PA contrast within NIR-I. It is noteworthy that this DDS effectively circumvent to toxicity of copper when applied in vivo, thereby suggesting a potential role for copper as a cost-effective material in future LNM imaging [196].

### Multimodality imaging

Although each of the above-mentioned imaging methods has unique advantages, they also possess certain defects that limit the ability to obtain reliable and accurate information. Combining two or more imaging modes into one system can produce more accurate imaging details than traditional imaging methods. An ideal strategy to enhance the accuracy and specificity of cancer diagnosis has emerged by coupling various LN imaging modalities, such as NIR fluorescence imaging, US imaging, and PA imaging. This multimodal imaging strategy yields a more comprehensive representation of the lymphatic system, facilitating the detection of a greater number of anomalies. Consequently, doctors can devise more efficacious treatment plans for cancer patients by integrating multiple imaging technologies.

The aforementioned enhancement of PA/US imaging through the utilization of carbon NP-based DDSs exemplifies this integrated imaging approach. Certain researchers have amalgamated the high specificity of PA imaging with the high sensitivity of fluorescence imaging to counteract the limited imaging depth of fluorescence imaging and the reduced sensitivity of PA imaging [197]. Serving as a “bridge” between two distinct imaging techniques, the gold nanocluster with PEGylated chitosan coating and ICG mixture (GC-AuNCs/ICG) in the DDS plays a role in the combined imaging technology, operating synergistically and without mutual interference, thereby leveraging the benefits of both imaging techniques [198].

In another study, researchers employed a DDS to deliver PA and fluorescence dual-modal nanoprobes for experimental studies on mouse models of breast cancer [199]. The high spatial resolution and depth of 3D information supplied by preoperative PA imaging were utilized to locate the primary lesion and guide tumor excision. During the procedure, the high sensitivity of NIR fluorescence imaging was harnessed to map suspected residual lesions and metastatic LNs in the vicinity of the surgical area for secondary resection [200]. Pathological analysis post-surgery revealed that over 70% of the secondary resection specimens confirmed the presence of tumor cells with the aid of fluorescence imaging. Following surgery guided by PA/fluorescence combined imaging technology, the local recurrence rate in the mouse model was 0 after 30 days, significantly lower than the 33.3% local recurrence rate observed in the control group.

Apart from PA/fluorescence combined imaging, Yang et al. coupled iron oxide with amino-terminal fragments (ATFs) to formulate a DDS for NIR/PA/MR combined imaging in mouse models of pancreatic cancer [201]. This DDS was capable of specifically delivering its contents to the target LN. In vivo combined imaging demonstrated that optical imaging can accurately locate the primary tumor and multiple metastases, and MRI providing high-resolution imaging of the lesions following localization, offering rich anatomical details [202]. Such high-resolution imaging data prove advantageous for surgeons, allowing for a detailed understanding of the lesions and avoidance of damage to adjacent tissues. The specific binding of the targeted multimodal imaging mode enhances the selective accumulation of the imaging agent at the target, making the diagnosis more accurate. The evolution and advancement of DDS-based multimodal imaging modes have significantly contributed to the localization of LN metastases, reduced surgical trauma, and generated novel insights for the future progression of LN imaging technology [203].

## Integration of diagnosis and treatment in DDSs

Post precise diagnosis, surgical resection stands as the primary treatment modality for cancer and LNM, with radiotherapy and chemotherapy acting as supplementary or adjunctive therapies during the treatment process. Traditional radiotherapy and chemotherapy, however, lack specificity and may engender adverse effects on normal tissues, thereby impacting overall health [204]. Substantial research has indicated that DDSs, owing to their high targeting and customization capabilities, are efficacious in delivering imaging agents or therapeutics [164, 205–210]. Hence, researchers have endeavored to amalgamate the diagnostic and therapeutic functions of DDSs.

**Fig. 4:**
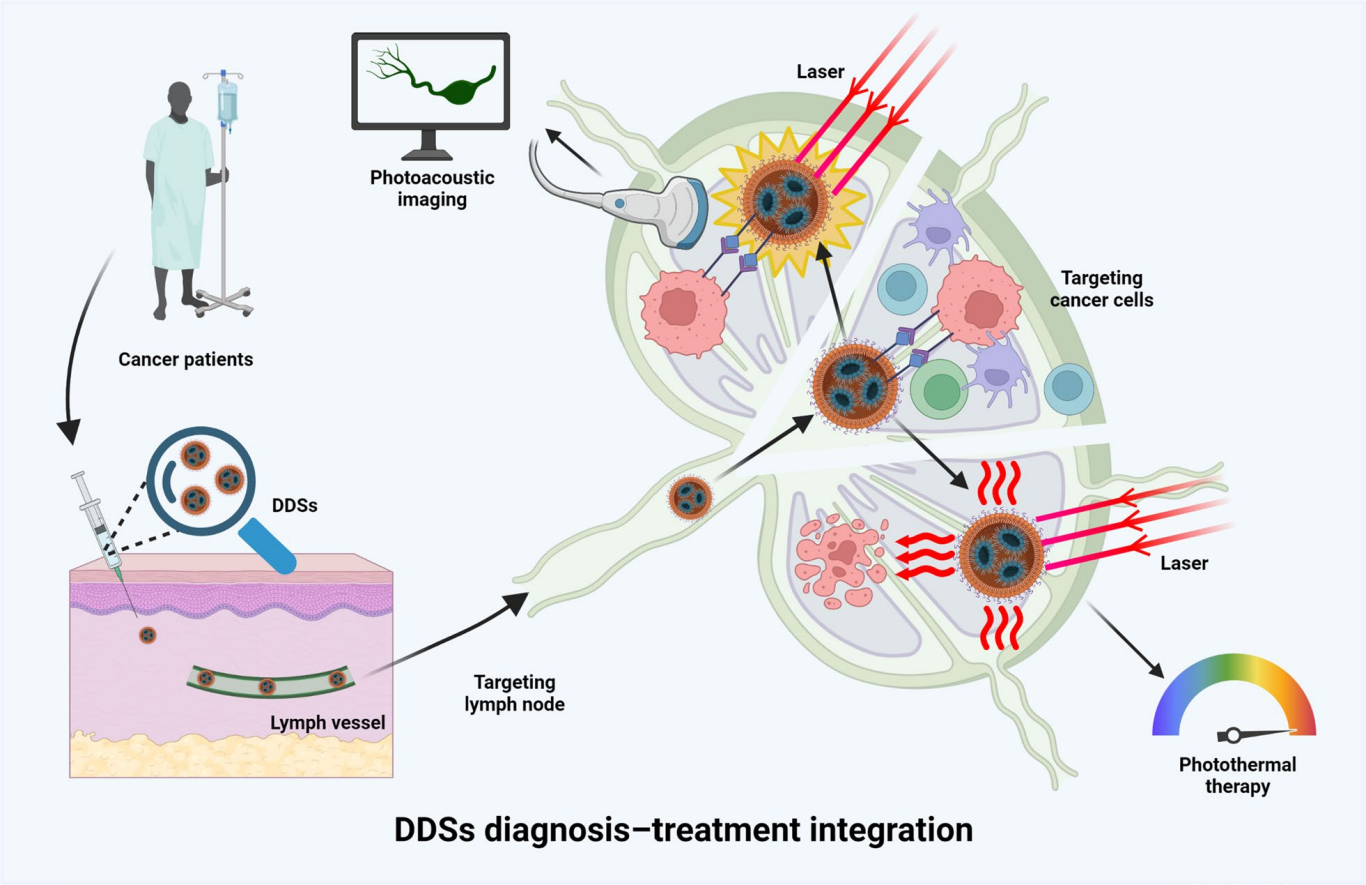
The dual role of drug delivery systems (DDSs) in diagnosis and treatment, demonstrating the application of DDSs in delivering photothermal agents. These agents convert light energy into photoacoustic signals for imaging, while simultaneously releasing thermal energy, derived from light absorption, to annihilate tumor cells. Created with BioRender.com

Among the multitude of integrated diagnosis-treatment approaches, photothermal therapy (PTT) has been garnering considerable attention. Given that tumor cells exhibit lower heat tolerance than normal cells, PTT capitalizes on this property to eradicate cancer cells by generating heat energy through the photothermal conversion of photothermal agents under irradiation at specific wavelengths [211]. Prussian blue DDSs, with their excellent biocompatibility, photothermal conversion efficiency, and absorption range within the NIR window, prove to be an optimal choice for PTT and PA imaging. Upon laser exposure, Prussian blue DDSs convert light energy into PA signals for PA imaging, while concurrently releasing the thermal energy converted from light absorption to exterminate tumor cells [212]. Nevertheless, this thermal ablation treatment modality lacks targeted capability towards lesion areas, and may induce damage to adjacent tissues if the temperature is excessively high [213], thereby diminishing patients’ quality of life. This shortcoming limits the clinical application and therapeutic efficacy of PTT; hence, it is necessary to improve the targeting of PTT and reduce the side effects of treatment [214]. Building on the high expression of the CD44 molecule in cancer cells [77], researchers have coated the CD44 ligand, hyaluronic acid (HA), onto DDSs loaded with chemotherapeutic drugs. This strategy actively transports diagnostic and therapeutic agents by targeting cancer cells rich in CD44, thereby significantly enhancing the targeting of imaging agents and decreasing the damage of PTT to the surrounding tissue [214]. Such a DDS achieves the integration of diagnosis and treatment for LNM by concurrently enabling imaging, PTT, and targeted transport of chemotherapeutic drugs under 1000–1700 nm second NIR window laser exposure. Initial drug delivery by DDSs is marked by a clear and stable NIR imaging signal; as PTT and chemotherapy drugs gradually inflict damage to tumor cells, the NIR signal significantly diminishes, which further confirmed its effect (Fig. 4).

Apart from PTT and the delivery of conventional chemotherapy drugs, DDSs can also actualize diagnostic and therapeutic effects in other ways. For instance, Yang et al. synthesized a high-affinity ligand for the urokinase plasminogen activator receptor. When coupled with a carrier, this ligand can inhibit tumor growth by competitively blocking the binding of natural ligands to receptors while also imaging regional LNM in pancreatic cancer [201]. Additionally, research on nano-microbubbles-based DDSs in integrated diagnosis-treatment approaches has also garnered interest. Utilizing US waves, microbubble oscillation is induced to enhance the ultrasonic contrast of the target site. Gases present in microbubbles, such as dioxygen, NO, and CO, can modify the tumor microenvironment, thereby generating therapeutic effects [215–217]. There is no doubt that although surgery is the main treatment for LNM, the concept of integrating diagnosis and treatment through DDSs provides a new idea for the minimally invasive and precise treatment of LNM. The diagnostic and therapeutic model based on DDSs harbors immense potential for clinical application.

## Challenges and prospects

**Fig. 5:**
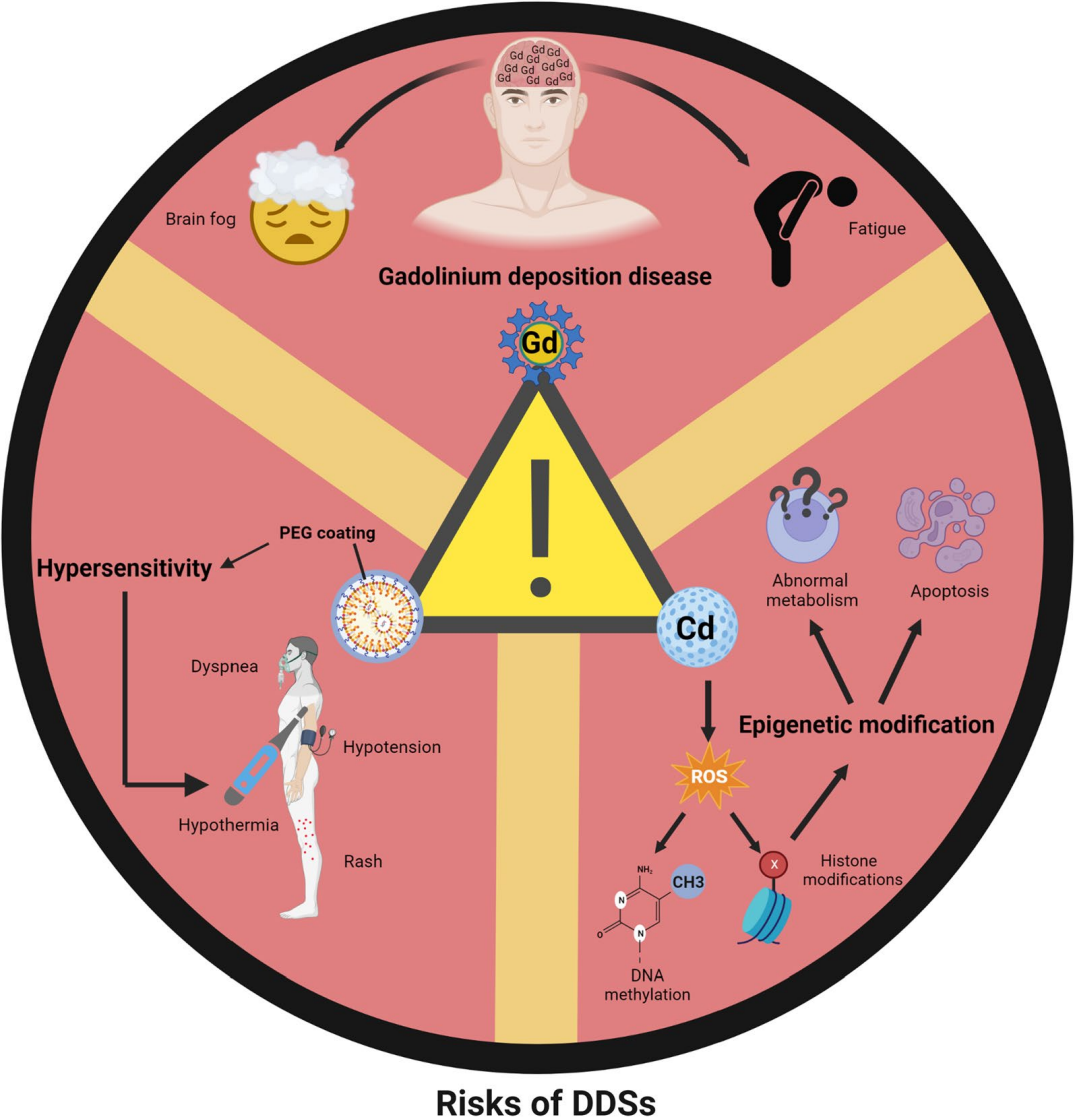
The potential risks associated with drug delivery systems (DDSs) in the context of lymph node metastasis imaging. Nanoparticle (NP)-based DDSs are not devoid of safety concerns. For instance, DDSs containing Gd may precipitate gadolinium deposition disease, causing symptoms such as fatigue and brain fog. Quantum dot-based DDSs may induce the production of reactive oxygen species (ROS) via the release of metal ions, such as the Cd ion, resulting in epigenetic modifications. Despite its initial status as an ideal, safe coating to mitigate DDS side effects, polyethylene glycol (PEG) might still instigate hypersensitivity reactions. Created with BioRender.com

As previously delineated, LN imaging predicated on DDSs may alleviate the toxic side effects of traditional contrast agents and enable the targeted transport of chemotherapy drugs within an integrated diagnosis and treatment model, thus reducing damage to normal cells [106, 122, 218]. The development and research of DDSs have advanced significantly; however, the potential for clinical transformation of DDSs remains limited. Although the global nanomedicine market was valued at $242.6 billion, with 563 nano-based DDS products in various stages of clinical trials or development in 2021 [219], only about 100 nanomedicines have actually been commercialized. Factors such as toxicity, high costs, and unclear regulatory guidelines have emerged as substantial barriers to their clinical application [220]. The risks associated with DDSs predominantly pertain to metabolic and toxicity factors, as well as a lack of standardization, presenting challenges for the application and transformation of DDSs (Fig. 5).

### Metabolism of DDSs

Typically, upon systemic introduction, DDSs accumulate, albeit to a certain extent, in various anatomical structures such as LNs, heart, spleen, and kidneys via fluid distribution, and are subsequently excreted through diverse pathways, thus avoiding significant bodily accumulation [221–223]. Contrarily, certain studies have found that some DDSs may persist within the body and prove challenging to effectively eliminate, thereby engendering side effects [224, 225]. Not only in the brain, but studies have also found that NPs can pass through biological barriers, deposit in the reproductive system, and cause damage to germ cells [226–228]. Studies have shown that plasma proteins adsorb onto the surface of NPs, altering their properties (such as size, shape, surface charge) and leading to abnormal protein aggregation and folding. This subsequently causes the NPs to off-target, reducing drug utilization, and leading to accumulation [229]. The development and use of modified coatings, such as PEG, will be very helpful. In addition, the metabolic characteristics of common abnormal accumulation organs such as the kidneys, liver, and brain need to be further studied, and the interactions between DDSs and these organs need to be explored.

### Toxicity of DDSs

Despite the great strides made in biosafety, DDSs still pose hidden dangers of toxicity [230]. DDSs based on quantum dots can stimulate the production of reactive oxygen species (ROS) through the release of metal ions, such as Cd ions [231]. This process can disrupt cellular metabolism [232], inflict DNA damage [233], and ultimately precipitate cell apoptosis [234]. Furthermore, the pro-oxidation properties of DDSs may elicit epigenetic alterations by interfering with RNA and chromatin remodeling [235]. Examples of such interactions include gold NPs influencing the activity of histone deacetylase [236] and superparamagnetic iron inducing high acetylation of core histones [237].

To imbue DDSs with safety, inertness, and stability, the carrier portion can be modified via PEGylation. PEG is considered a non-immunogenic material, which not only mitigates the interaction between the DDS core and the biological milieu, thereby enhancing safety [171, 238], but also bestows DDSs with stealth effects, diminishes protein adsorption, and precludes cellular ingestion, thus extending circulation time. Therefore, PEG was once widely considered an ideal and safe strategy to reduce the toxicity of DDSs [239–242]. Regrettably, a small amount of evidence suggests that PEG can trigger hypersensitivity reactions [243–245], manifesting symptoms such as respiratory distress, hypothermia, hypotension, rashes, and even mortality [246], thereby jeopardizing patient safety. Furthermore, it has been proven that the combination of PEG with protein or lipid NP materials can induce the body to produce anti-PEG antibodies, which often affect the distribution of PEGylated products and enhance their clearance rate, thus affecting the realization of the expected efficacy [247–250]. With the increasing application of PEG in cosmetics, food, medicine, vaccines, and other fields, the incidence of hypersensitivity reactions and the reduction of the efficacy of PEGylated drugs may increase [251]. At present, the mechanism of PEG-based DDS-induced hypersensitivity and anti-PEG antibodies is still uncertain. However, due to the widespread use of PEG, understanding its mechanism and solving adverse reactions is very important to obtain more effective preventive measures. Further development of modified coatings with low or no immunogenicity without compromising performance is warranted.

### Delivery mechanism of DDSs

To achieve superior image quality, DDSs must rapidly infiltrate and accumulate in metastatic LNs. Despite ongoing research and innovation, the delivery efficiency of extant DDSs remains suboptimal. A retrospective analysis showed that only 0.7% (median) of the administered dose was delivered to solid tumors [252]. Previous studies have suggested that the tumor accumulation of DDSs is highly dependent on their size, based on the EPR effect [155, 253, 254]. As previously noted, excessively large DDS particle sizes impede LN entry, while exceedingly small particle sizes limit metastatic LN retention [154]. However, some studies have questioned the effectiveness of the EPR effect in the human body [255, 256]. Currently, there is no consensus on whether the EPR effect exists in the human body or the appropriate size range for DDS carriers [159, 257]. In the future, it is necessary to verify the effect of EPR, focusing on exploring the accumulation, isolation, and clearance mechanisms of DDSs in the human body, as well as the interaction between DDSs and tumors, in order to further improve the delivery efficiency of DDSs.

### Specificity of DDSs

To minimize side effects and extraneous organ distribution, DDSs should exhibit enhanced targeting of tumors and metastatic LNs. The existing targets for tumors and LNM do not fully meet the needs of accurate imaging, posing a hindrance to the clinical application of DDSs. The development of additional immune targets to augment the specificity of LN imaging, based on tumor characteristics and the metastatic LN microenvironment, might emerge as a prominent future direction [258–260]. With advancements in sequencing technology, clinical precision medicine has been greatly promoted [261]. Multi-omics analyses, such as genomics, metabolomics, and transcriptomics, are important means to identify therapeutic targets and molecular biomarkers currently and in the future, and to realize the personalization of cancer treatment [261–263]. In addition, the deep learning model based on radiomics and digital pathology also holds far-reaching significance for the mining of immune targets and imaging diagnosis [264].

### Integration of diagnosis and treatment of DDSs

DDSs represent a method of administering imaging agents imbued with boundless potential. In the medical realm, DDSs have robustly validated their value in both imaging and drug delivery. Concurrent with the evolution of nanotechnology, the concept of “diagnosis-treatment integration” through DDSs has been proposed. This concept is capable of executing therapeutic functions while accurately locating lesions or metastatic LNs. The previously mentioned PA/PTT technology constitutes an initial realization of this concept. Nevertheless, it is important to note that multimodal imaging still has many shortcomings that need to be addressed by technological development, such as high cost, cumbersome imaging procedures, and the burden of high doses of imaging agents on the patient. Looking forward, we anticipate that DDSs can fully actualize their highly customized potential, achieving multifunctionality not only for diagnosis-treatment integration but also in becoming a “universal imaging agent and delivery system.” As a conduit linking various imaging technologies with their respective advantages, DDSs can enable the synergistic functioning of imaging technologies without burdening patients with the risk of multiple imaging agent injections.

### Standardized risk assessment protocols

The dearth of standardized risk assessment protocols for DDSs constitutes a significant issue warranting collective attention, which may lead to patients receiving inappropriate treatment or missing the best treatment opportunity [265]. Regarding risks, researchers and clinicians may need to comprehensively explore the potential risks of DDSs applied to LN imaging, stratify these risks according to their likelihood and severity, and then establish a standardized treatment plan based on the corresponding risk stratification. Only by addressing these fundamental problems can we effectively promote the development and clinical transformation of DDSs. It is important to note that the Cancer Nanomedicine Repository provides a database that can determine the relationship between the physical and chemical properties of biological systems and NPs in real time, and analyze the delivery efficiency of DDSs [266]. Sharing and continuously analyzing experimental data is of positive significance for the clinical transformation of DDSs.

Such issues have considerably impeded the clinical adoption of DDSs. There is a need to promote the development of research, overcome the limitations of existing technologies, and develop DDSs that can effectively achieve the dual goals of reducing toxicity and increasing efficiency. Then, multi-stage preclinical and clinical trials should be performed. On the premise of ensuring biosafety, reducing costs and improving availability are necessary. The clinical transformation of the technology can ultimately be realized through the approval of relevant regulatory authorities.

## Conclusions

The use of DDSs for imaging LNs presents an innovative approach in diagnosing LNM. The application of DDSs in LN targeting addresses several drawbacks associated with traditional, non-targeted drug delivery methods, notably by enabling precise localization within LNs. This precision enhances both the effectiveness and control of imaging agent delivery through multiple pathways. Consequently, it not only elevates the concentration of therapeutic agents in affected tissues but also significantly enhances the diagnostic accuracy for metastatic LNs. At the same time, this method allows for a reduction in the required effective drug dosage, thereby diminishing potential side effects [220]. Moreover, the coatings of DDSs can encapsulate imaging agents within their cores, thus precluding reactions with the biological environment and ameliorating biological distribution. This reduces bodily accumulation and consequently diminishes the toxicity of imaging agents. Hence, employing DDSs to target LNs for imaging represents a nascent, promising technology. Although it necessitates further refinement and development for utilization in LNM diagnosis, the application of DDSs significantly enhances the accuracy of diagnosis, improves the success rate of surgery, reduces the incidence of surgical complications, and improves the quality of life of patients. There is no doubt that employing DDSs is an ideal LNM imaging.

## Data Availability

No datasets were generated or analysed during the current study.

## Abbreviations

APC: Antigen-presenting cell
ATF: Amino-terminal fragment
CT: Computed tomography
DC: Dendritic cell
DDS: Drug delivery system
EPR: Enhanced permeability and retention
EMA: European Medicines Agency
FDA: Food and Drug Administration
GBCA: Gadolinium-based contrast agent
HA: Hyaluronic acid
ICG: Indocyanine green
LYVE-1: Lymphatic vessel endothelial hyaluronan receptor-1
LN: Lymph node
LNM: Lymph node metastasis
LSG: Lymphoscintigraphy
MRI: Magnetic resonance imaging
NIR: Near-infrared
NIR-I: The first NIR window
NIR-II: The second NIR window
NP: Nanoparticle
PA: Photoacoustic
PET: Positron-emission-tomography
PSMA: Prostate-specific membrane antigen
PLGA: Poly (d,l-lactic-co-glycolic acid)
PCD: Polycyclodextrin
PEG: Polyethylene glycol
ROS: Reactive oxygen species
RES: Reticular endothelial system
SPECT: Single-photon-emission-computed-tomography
SLN: Sentinel lymph node
SPR: Surface plasmon resonance
US: Ultrasound
USPIO: Ultrasmall superparamagnetic particles of iron oxide
PTT: Photothermal therapy
99mTc: Technetium-99
18F-FDG: 18F-Fluorodeoxyglucose
γ-PGA: Poly (γ-glutamic acid)
